# High tuberosity healing rate associated with better functional outcome following primary reverse shoulder arthroplasty for proximal humeral fractures with a 135° prosthesis

**DOI:** 10.1186/s12891-020-3060-8

**Published:** 2020-01-16

**Authors:** Jonas Schmalzl, Malik Jessen, Nadine Sadler, Lars-Johannes Lehmann, Christian Gerhardt

**Affiliations:** 1 Department of Traumatology and Hand Surgery, St. Vincentius Clinic, Karlsruhe, Academic Teaching Hospital Albert-Ludwigs-University Freiburg, Suedendstraße 32, D-76137 Karlsruhe, Germany; 2 Medical Faculty Mannheim, Karls-Ruprecht-University Heidelberg, Mannheim, Germany

**Keywords:** Humeral inclination, 135, Tuberosity healing, Proximal humeral fracture, Reverse shoulder arthroplasty

## Abstract

**Background:**

Reverse shoulder arthroplasty (RSA) is a common treatment for proximal humeral fractures. (PHF) in the elderly. This study evaluates the functional outcome and the influence of. tuberosity healing (TH) following RSA with 135° humeral inclination and a neutral glenosphere without lateralization for PHFs.

**Methods:**

In this retrospective case series, all patients with an acute PHF treated with primary RSA with 135° humeral inclination and a standard glenosphere without lateralization during a four-year period were followed up. Constant score (CS), patient satisfaction (subjective shoulder value (SSV)), TH and glenoid notching were analyzed.

**Results:**

38 patients with a mean age of 77 ± 8 years were available for follow-up at 34 ± 5 months. The mean adjusted CS was 61 ± 9 points. TH of the greater tuberosity (GT) was 82% and resulted in significantly improved abduction (117° vs. 81°; *P* < 0.001), forward flexion (139° vs. 99°; *p* < 0.001), external rotation (28° vs. 10°; *p* = 0.002), CS (65 vs. 41 points; *p* < 0.001) and patient satisfaction (SSV 79% vs. 48%; p < 0.001). TH of the LT was 87% without affecting internal rotation or overall outcome. The complication- and revision rate was 5%; implant survival was 100%. Scapular notching occurred in 3 (8%) cases (all grade 1).

**Conclusion:**

RSA with 135° humeral inclination and a standard glenosphere for PHF leads to good functional outcome in combination with a high rate of TH and a low rate of scapular notching. The short-term revision rate is low and the results are predictable and continuous. TH is associated with improved ROM, patient satisfaction and functional outcome.

## Introduction

Proximal humeral fractures (PHF) account for 5% of all fractures [1, 2]. Avascular necrosis of the humeral head after trauma or after osteosynthetic treatment of PHF represents a major problem of joint-preserving therapy approaches [1, 3–5]. Especially in the elderly, the results after osteosynthesis might not be satisfactory and revision rates up to 25% are reported [6]. Due to decreased bone quality, comminution and displacement as well as the aim to decrease revision surgery, arthroplasty is commonly used to treat PHF in elderly with a high risk for vascular compromise. However, functional outcome after hemiarthroplasty (HA) for acute PHF in patients older than 70 years have been disappointing and unpredictable [7–10]. This is mainly attributed to poor bone quality associated with low tuberosity healing (TH), severe comorbidities and incompliance to the rehabilitation protocol [11]. After the development of reverse shoulder arthroplasty (RSA) by Paul Grammont [12] several studies have demonstrated that functional outcome after RSA for acute PHF in such a cohort are superior to HA [7, 9, 13]. However, the impact of TH on function after RSA has only received limited study. Furthermore, it is unclear if certain design features of a given RSA like the humeral inclination angle affect the clinical outcome and GT healing rate.

The aim of this study was to evaluate functional outcome and influence of TH following RSA with 135° humeral inclination and a standard glenosphere without lateralization or inferiorization for PHFs. The hypothesis was that TH would lead to improvement in functional outcome.

## Materials and methods

A single center retrospective case series of all PHFs treated with RSA during a four-year period was performed. Institutional review board approval was obtained. All patients signed informed consent and gave their approval for the use of clinical and radiographic data for scientific purposes.

Inclusion criteria:

- Surgical treatment between January 1, 2012 and December 31, 2016
- An acute PHF defined as treatment within 6 weeks of injury
- Minimum clinical and radiographic follow-up of 24 months

Exclusion criteria:

- Previous surgery of the involved shoulder
- Patients with severe neurological disorders unable to follow the postoperative management regime
- Revision shoulder arthroplasty

### Surgical technique

**Fig. 1:**
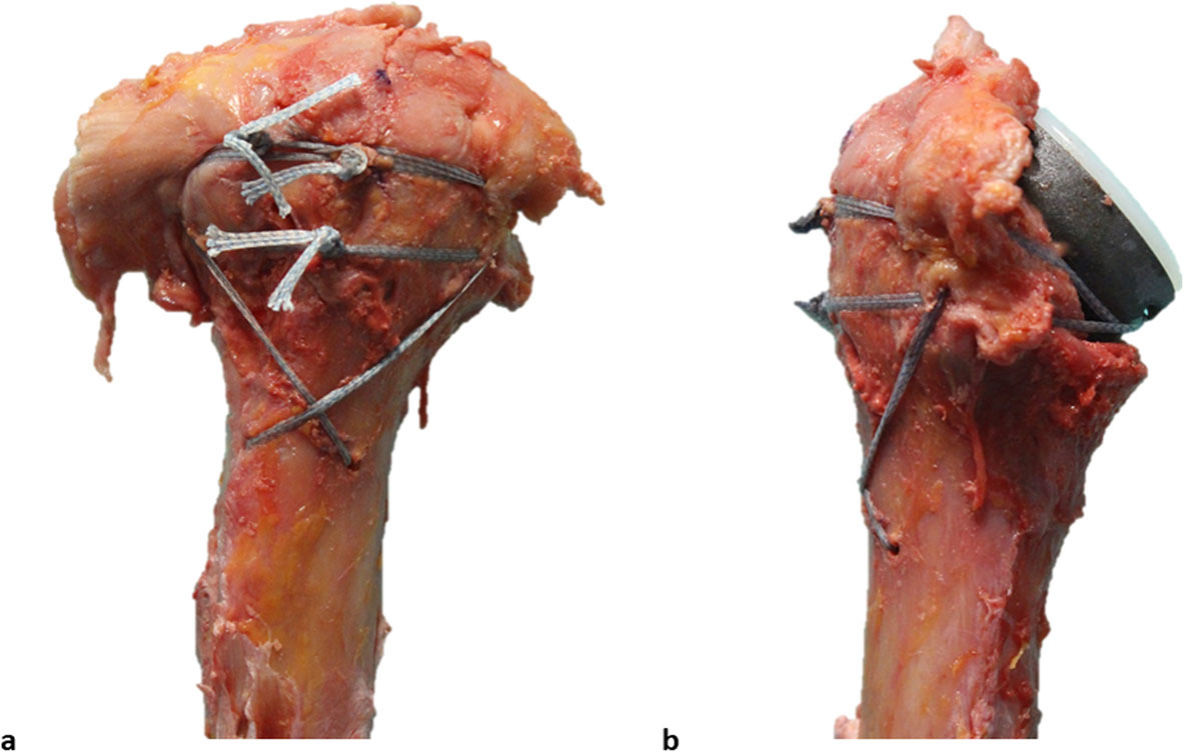
Technique of tuberosity repair. The tuberosity fragments are reconstructed horizontally around the neck of the prosthesis with two suture cerclages. These go through a hole at the medial neck of the prosthesis and the subscapularis and infraspinatus tendons. In the vertical plane, a cerclage in a figure-of-8 technique reduces the tuberosities to the shaft **a, b**

Preoperative X-rays in 2 planes (anterior-posterior (AP) and Y-view) and a computed tomography (CT) scan were obtained. All patients underwent surgery in beach chair position under general or regional anesthesia. Surgery was performed by one single surgeon (LL). A deltopectoral approach was performed in all cases using a consistent implant system (Univers Revers; Arthrex, Naples, USA). The humeral stem was cemented in 19 cases (50%) and placed in a press-fit fashion in 19 cases (50%) depending on the bone quality. In this cohort in all cases a humeral inclination angle of 135° was selected. Tenotomy of the long head of the biceps was routinely performed. Following identification of the tuberosities and resection of the humeral head, a baseplate was placed on the glenoid. In all cases a standard glenosphere without lateralization or inferiorization was selected. The glenosphere size was chosen depending on the patient’s anatomy: 36 mm in 10 cases, 39 mm in 26 cases and 42 mm in 2 cases. Following fixation of the humeral stem, the tuberosities were horizontally sutured around the stem of the prosthesis with a double suture cerclage (Fibertape, Arthrex). An additional vertical suture was applied between humeral shaft and prosthesis in a figure-of-eight configuration in all cases. The cerclage technique is illustrated in Fig. 1.

Postoperatively the shoulder was immobilized in an abduction pillow for 6 weeks. Passive range of motion was initiated after 3 weeks. The sling was removed after 6 weeks and active range of motion was allowed. Strengthening began 12 weeks postoperatively.

### Postoperative Evaluation.

The patients were asked to grade pain on a visual analogue scale (VAS). Active range of motion (ROM) was measured with a goniometer for elevation, abduction, and external rotation of the elbow at the side. Internal rotation was judged by the level of vertebra reached by the thumb and was graded with a numeric ordinal scale. Functional outcome was assessed using the Constant score (CS) as shoulder specific score. In addition, the Subjective Shoulder Value (SSV) was used as patient-focused outcome tool.

Radiographic assessment at follow-up was performed by one examiner (JS) based on an AP view in neutral rotation and an axial view. Greater and lesser TH was assessed as yes or no. When it was in an anatomical position on the axial view and visible on the AP X-ray the GT was considered to be healed. The LT was evaluated on the axial view. Scapular notching was evaluated in the AP view according to Sirveaux [14].

### Statistical analysis

Statistical analysis was performed with SPSS version 22 (IBM, Armonk, USA) using the independent samples Mann-Whitney U-test and the Kruskal-Wallis test. Quantitative variables were described by means, standard deviations, minimums and maximums. Normal distributions were tested by the Shapiro-Wilk test and confirmed graphically by histogram. In order to determine prognostic factors for the functional outcome correlation between ROM, VAS, SSV, CS and the following parameters were tested: TH, fixation technique of the stem (cemented vs. press fit) and age. *P* values ≤0.05 were considered to be significant.

## Results

58 patients met the inclusion criteria. 13 (20%) were deceased from unrelated causes and 7 (11%) were lost to follow-up leaving 38 patients available for follow-up at a mean of 34 ± 5 months after surgery. The mean age was 77 [62–97] years at the time of surgery. All fractures were classified according to the Neer [15] and the OTA [16] classification. Baseline characteristics are summarized in Table 1.

**Table 1:** Baseline characteristics. *SD* standard deviation

*Variable*	
*n*	38
Mean patient age in years [SD]	77 [±8]
Mean follow-up in months [SD]	34 [±5]
Gender	
Men	5 (13%)
Women	33 (87%)
Injured side	
Right	26 (68%)
Left	12 (32%)
Neer Classification	
Type I (1-part)	0
Type II (2-part)	0
Type III (3-part)	5 (13%)
Type IV (4-part)	33 (87%)
Humeral fixation	
Press fit	19 (50%)
Cemented	19 (50%)
OTA/AO Classification	
B2	5
C2	31
C3	2

Mean postoperative abduction was 111° ± 20°, mean forward flexion was 132° ± 22° and mean external rotation at the side was 25° ± 13°. Mean internal rotation was at vertebra L5. The mean CS was 61 [24–80] points, the mean age-adapted CS was 74 [30–99] percent and the mean SSV was 74 [20–100] percent. Average pain level on the VAS was 1 ± 1 out of 10 points. Patient outcomes are outlined in Table 2.

**Table 2:** Postoperative outcome. *SD* standard deviation; *L5* lumbar vertebra 5

*Outcome parameter*	
Mean Constant score [range]	61 [24–80]
Mean adjusted Constant score [range]	74 [30–99]
Mean Subjective Shoulder Value score [range]	74 [20–100]
Mean pain level on Visual Analogue Scale [SD]	1 [±1]
Mean abduction [range]	111° [±20°]
Mean forward flexion [range]	132° [±22°]
Mean external rotation at 0° [range]	25° [±13°]
Mean internal rotation	L5
Complications	2 (5%)
Revision surgery	2 (5%)
Implant failure	0
Greater tuberosity healed	31 (82%)
Lesser tuberosity healed	33 (87%)
Inferior notching according to Sirveaux	8%
- grade 1	3
- grade 2	0
- grade 3	0
- grade 4	0

The overall healing rate of the GT was 82%. GT healing significantly improved CS (65 vs. 41 points; *p* < 0.001), patient satisfaction (SSV 79% vs. 48%; *p* < 0.001), abduction (117° vs. 81°; *P* < 0.001), forward flexion (139° vs. 99°; p < 0.001) and external rotation (28° vs. 10°; *p* = 0.002). The difference in functional results between patients with anatomic GT healing and those in whom it was resorbed are summarized in Table 3. Interestingly, all patients except one (86%) who presented GT resorption were females.

**Table 3:** Outcome according to greater tuberosity healing. *ADL* activities of daily living; *GT* greater tuberosity; *L4* lumbar vertebra 4; *n* number; *n.s.* not significant; *S1* sacral vertebra 1; *SD* standard deviation

Variable	GT healed	GT not healed	*p*-value
*n*	31	7	
Age at surgery in years [SD]	77 ± 10	76 ± 11	n.s.
Constant Score in points [SD]	65 ± 7	41 ± 11	< 0.001
Pain [SD]	14 ± 2	10 ± 5	0.001
ADL [SD]	17 ± 3	12 ± 3	< 0.001
Mobility [SD]	29 ± 4	16 ± 5	< 0.001
Strength [SD]	6 ± 3	2 ± 2	0.003
Subjective Shoulder Value [SD]	79% ± 11	48 ± 20	< 0.001
Active abduction	117° ± 23°	81° ± 15°	< 0.001
Active forward flexion	139° ± 21°	99° ± 28°	< 0.001
Active external rotation	28° ± 15°	10° ± 10°	0.002
Active internal rotation	L4	S1	n.s.

**Fig. 2:**
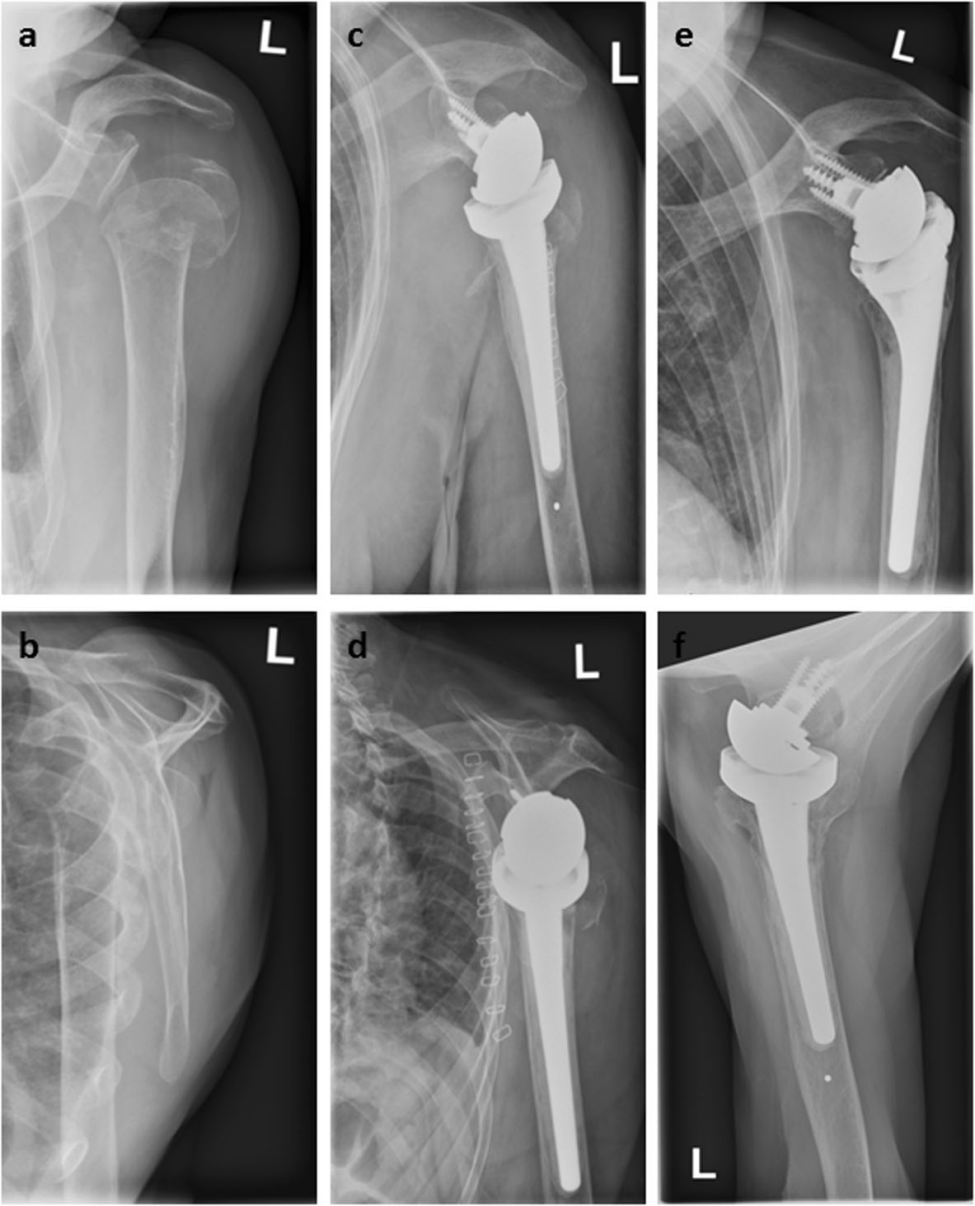
Pre- **a, b**, postoperative **c, d** and follow-up images **e, f** of a 97-year-old patient with a 11-C2-fracture of the proximal humerus who was treated with primary reverse shoulder arthroplasty. At final follow-up after 24 months the Constant Score was 68 points. Both lesser and greater tuberosity are healed in an anatomical position **e, f**

**Fig. 3:**
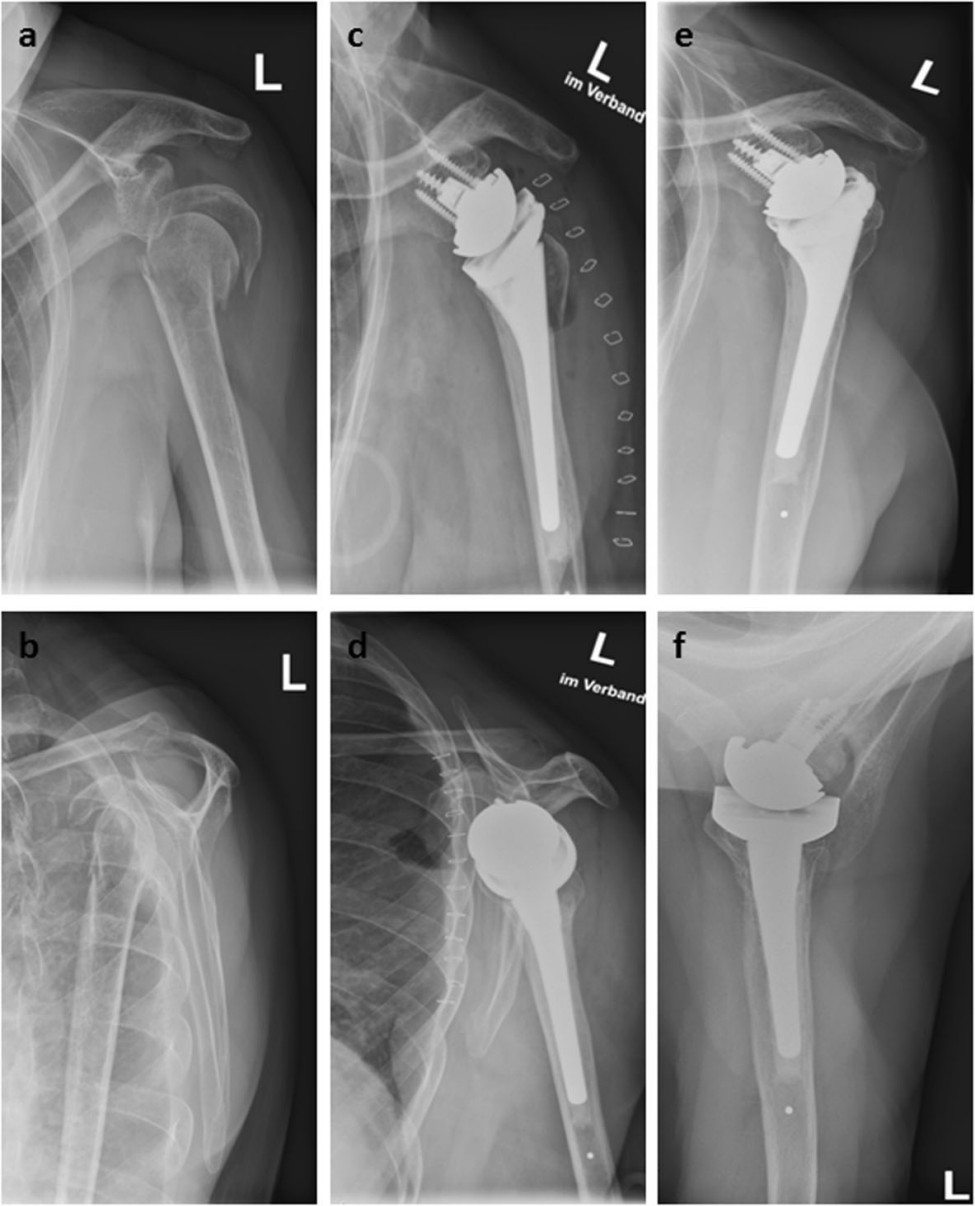
Pre- **a, b**, postoperative **c, d** and follow-up images **e, f** of a 68-year-old patient with a 11-C2-fracture of the proximal humerus who was treated with primary reverse shoulder arthroplasty. At final follow-up after 36 months the Constant Score was 51 points. The lesser tuberosity healed in an anatomic position; however, the greater tuberosity was resorbed **f**

Two exemplary cases with and without GT healing are illustrated in Fig. 2 and Fig. 3.

Overall LT healing was 87%, however, it did not affect internal rotation or functional outcome.

Regarding humeral fixation technique (cemented vs. press-fit) no statistical differences could be observed.

Patient age did not negatively correlate with the outcome.

Inferior notching occurred in 3 cases (8%), of which all 3 were grade 1.

The complication and revision rate was 5%. One case presented with a postoperative hematoma which had to be surgically addressed. Another patient suffered an atraumatic dislocation which was treated with revision surgery and a metaphyseal extension of 6 mm was additionally implanted; no further dislocation occurred. No prosthesis required removal during the study period.

## Discussion

Multiple studies unanimously showed good clinical short- to midterm results and a low complication and revision rate after RSA for acute PHF in the elderly [7, 9, 13, 17–21].

It is well established that bony healing of the tuberosities in an anatomical position is the most important single factor for a good clinical outcome after primary implantation of a HA for fracture [4, 10, 22]. Resorption or dislocation of the tuberosities leads to dysfunction of the rotator cuff and significant functional limitations [8, 10, 15].

In contrast to the results from HA, the need and potential benefits of tuberosity refixation in case of RSA remain controversial as the deltoid muscle can partially compensate for the loss of internal and external rotation, caused by rotator cuff deficiency or tuberosity resorption, through medialization and caudalization of the fulcrum. To date, TH rates between 37 and 84% have been reported in the literature in RSA for acute PHF in the elderly population [7, 9, 13, 17–21]..

Several recent studies have examined the influence of GT healing on functional outcome after RSA for acute PHF, therefore, we compared our results with the available literature as shown in Table 4.

**Table 4:** Comparison of studies evaluating outcome and influence of tuberosity healing after reverse shoulder arthroplasty in proximal humeral fractures in elderly. *ASES* American shoulder and elbow surgeons score; *CS* constant score; *GT* greater tuberosity; *No.* number; *n.r.* not reported

Study (year)	No. of patients	Mean follow up (months)	Implant type	GT healing rate	Overall outcome	Outcome GT healed	Outcome GT not healed	Scapular notching	Complication rate
*Cuff (2013) (6)*	24	30	135° (DJO Altivate)	83%	77 pts. (ASES)	78 pts. (ASES)	75 pts. (ASES)	0%	8%
*Gallinet (2013)* [8]	41	24	155° (DePuy Delta 3 [24] Tornier Aequalis [20] Zimmer Anatomical Reverse [9])	66%	n.r.	60 pts. (CS)	52 pts. (CS)	73%	10%
*Sebastiá Forcada (2014)* [12]	31	29	155° (Lima SMR)	65%	56 pts. (CS)	59 pts. (CS)	54 pts. (CS)	3%	6%
*Garofalo (2015)* [16]	87	27	155° (Tornier Aequalis Fracture)	75%	n.r.	n.r.	n.r.	1%	5%
*Grubhofer (2016)* [17]	51	35	155° (Zimmer Anatomical Reverse)	84%	62 pts. (CS)	65 pts. (CS)	50 pts. (CS)	63%	8%
*Chun (2017)* [20]	38	37	155° (Tornier Aequalis)	37%	n.r.	68 pts. (CS)	64 pts. (CS)	29%	0%
*Torrens (2018)* [19]	41	29	155° (Depuy Delta Xtend)	68%	61 pts. (CS)	61 pts. (CS)	61 pts. (CS)	15%	2%
*Boileau (2019)* [18]	37	36	155° (Tornier Aequalis Fracture)	84%	64 pts. (CS)	64 pts. (CS)	51 pts. (CS)	47%	5%
*Current study*	38	34	135° (Arthrex Univers Reverse)	82%	61 pts. (CS)	65 pts. (CS)	41 pts. (CS)	8%	5%

(Table 4 should appear here in production of the manuscript)

The results of this study confirm our hypothesis that functional outcome is improved following RSA for PHF when GT healing occurs. TH was associated with superior functional outcome and higher patient satisfaction compared to when the tuberosity did not heal.

In contrast to our findings, Torrens et al. [20] reported a cohort of 41 patients with PHF treated with a primary 155° RSA at an average of 29 months. The GT healing rate was 68% but TH did not affect the CS. Chun et al. [21] examined a cohort of 38 patients who underwent RSA with 155° for PHF. GT healing rate was only 37% and there were no statistical differences in the overall functional outcome regardless of TH. Nevertheless, external rotation was significantly better in the healed GT group (29° vs. 10°). In a randomized controlled trial comparing HA and RSA with 155° for PHF Sebastiá-Forcada et al. [13] reported that in the RSA group with 31 patients functional outcome was also irrespective of TH.

On the contrary, Gallinet et al. [23] compared patients treated with different 155° prosthesis with and without tuberosity repair. In this study, the patients with tuberosity fixation exhibited better CS and the patients with successful TH yielded better shoulder function. In addition, both Grubhofer et al. [18] and Boileau et al. [19] reported significantly better functional outcome and patient satisfaction in case of 155° RSA with successful TH.

Consistent with these findings, we believe our results demonstrate that TH should be an important goal after RSA for PHF. In particular, because successful TH after RSA not only improves functional outcome but also may contribute to the avoidance of complications. In case of failed GT healing or tuberosity resection, high rates of complications (up to 40%) have been reported in some RSA case series for PHF [24–28]. In a series of 30 acute fractures treated with RSA without tuberosity reattachment, Cazeneuve et al. [26] reported 2 cases of instability, 2 with implant loosening, 1 with infection, and 7 with proximal humeral bone lysis. Klein et al. [29] described 2 early infections and 2 dislocations in a case series of 20 PHF treated with RSA and excision of the tuberosities. Gallinet et al. [9] analyzed 24 patients without or with failed tuberosity repair and observed 2 infections and 1 anterior dislocation of the implant. We did observe one atraumatic dislocation in our series, interestingly, this also occurred in a patient with failed tuberosity repair. In our opinion the reattachment of the tuberosities and the adjacent rotator cuff tendons stabilizes the implanted prosthesis and guarantees better soft tissue coverage which minimizes the risk of infection. Nevertheless, there is a variety of other factors that may be attributed to the mentioned complications like surgical technique, patient associated factors (e.g. age, gender or osteopenia) and implant design.

As shown in Table 4, the GT healing rate is highly varying. These differences may be attributed to fixation techniques and the type of prosthesis used. Recent efforts to enhance TH after RSA in complex fractures include modifications in implant design like fracture specific humeral stems with large ingrowth surface [17, 19] as well as bone graft techniques [27]. Considering the literature, the highest GT healing rates (around 80%) were either achieved with a 135° prosthesis or with a 155° prosthesis with a specific fracture stem.

Cuff et al. [7] performed a prospective cohort study comparing HA with RSA for PHF in patients > 70 years. In this study, the DJO Reverse Shoulder Prosthesis (DJO Surgical, Austin, USA) with a humeral inclination of 135° was used. However, compared to the 135° prosthesis used in our study, the DJO RSA implicates more glenoid lateralization. The GT healing rate was 83% and is similar to the 82% we achieved. Similar GT healing rates with a 155° prosthesis were only reported by Grubhofer et al. [18] in a cohort of 51 patients using the Zimmer Reverse System (Zimmer Biomet, Warsaw, USA) with a fracture specific stem and Garofalo et al. [17] as well as Boileau et al. [19] with a combination of autologous bone grafting and a fracture specific stem (that incorporates a cancellous bone autograft). We suspect that RSA with a humeral inclination of 135° allows refixation of the tuberosities in a more anatomic position and therefore might result in decreased stress on the tuberosity repair compared to a 155° prosthesis, thus successful GT repair in RSA is more predictable without having to use a fracture specific stem. However, it is clear that there are several other factors that may affect tuberosity healing like e.g. the fixation technique (number and kind of sutures/cables) or the bone quality.

Another advantage of the 135° design is the reduced risk for scapular notching. In 2015, a systematic review taking into account 38 studies and 2222 shoulders was published comparing RSA with 135° and 155° humeral inclination: it was reported that 135° inclination resulted in significantly less scapular notching without a higher rate of complications or significant differences in functional results relative to 155° inclination [30]. Whereas Erickson et al. [30] compared RSA with 155° and 135° implants and a lateralized glenosphere (Altivate; DJO Surgical), we used a different RSA with 135° inclination (Univers Revers; Arthrex) without lateralized glenosphere. In our eyes, in PHF without preexisting cuff tear arthropathy or osteoarthritis, the joint line is not medialized, therefore, all prostheses were implanted without lateralization of the glenosphere. Lateralization of the glenosphere is an established measure to reduce scapular notching; however, even without lateralization, we observed a very low rate of scapular notching. These findings are consistent with those reported by Cuff. et al. who also used a 135° prosthesis. In contrast, scapular notching rates up to 73% [23] are reported for the 155° prosthesis (see Table 4). This also correlates with the experimental results of Oh et al. [31] who found that a 135° prosthesis does not impinge until an average of 12° degrees of adduction, whereas a 155° prosthesis will notch with the arm resting at the side. The impact of scapular notching on the clinical outcome remains controversial [14, 32, 33] but there is consensus that it should be avoided because of possible complications in the long term [34].

There are several limitations to the current study. First, our study has the inherent limitations of a retrospective series. Second, we did not compare the outcomes of 155° or 135° humeral inclinations as the surgeon in this study preferred the latter. While we believe our results support the use of a 135° prosthesis based on the high TH rate and the low occurrence of scapular notching, a randomized controlled trial may be needed to confirm these findings. Third, the follow-up is short- to midterm and could change over time.

## Conclusion

RSA with 135° humeral inclination and a neutral glenosphere for PHF leads to good functional outcome in combination with a high rate of TH and a low rate of scapular notching. The short-term revision rate is low and the results are predictable and continuous. TH is associated with improved ROM and functional outcome.

## Data Availability

The datasets used and/or analysed during the current study are available from the corresponding author on reasonable request.

## Abbreviations

AP: Anterior-posterior
CS: Constant score
GT: Greater tuberosity
HA: Hemiarthroplasty
LT: Lesser tuberosity
PHF: Proximal humeral fracture
RSA: Reverse shoulder arthroplasty
SSV: Subjective shoulder value
TH: Tuberosity healing
